# Peritoneal dialysis as a treatment option in autosomal dominant polycystic kidney disease

**DOI:** 10.1007/s11255-015-1087-9

**Published:** 2015-08-19

**Authors:** Magdalena Jankowska, Michał Chmielewski, Monika Lichodziejewska-Niemierko, Piotr Jagodziński, Bolesław Rutkowski

**Affiliations:** Department of Nephrology, Transplantology and Internal Medicine, Medical University of Gdańsk, ul. Dębinki 7, 81-211 Gdańsk, Poland

**Keywords:** Peritoneal dialysis, Polycystic kidney disease, Registry

## Abstract

**Purpose:**

When choosing a dialysis option for ADPKD patients, peritoneal dialysis (PD) is often discouraged, due to its potential drawbacks: risk of abdominal hernias and dialysis fluid leaks, risk of peritonitis and insufficient dialysis adequacy. The present study was designed to compare the outcomes and dialysis efficacy in ADPKD patients treated with PD, in comparison with non-ADPKD subjects.

**Methods:**

This study was a retrospective analysis of the data from the national PD registry in which 106 ADPKD and 1606 non-ADPKD incident PD patients were evaluated. Data on dialysis adequacy, risk of dialysis-associated complications, as well as patient and technique survival were compared between the groups.

**Results:**

The ADPKD patients did not differ from the non-ADPKD controls in terms of dialysis adequacy. After a median observation time of 32 months, there were no differences in patient or technique survival. The risk of abdominal hernias and dialysis fluid leaks was twice as high in ADPKD subjects, compared to the non-ADPKD group. However, these complications did not result in a need for a permanent transfer to hemodialysis.

**Conclusions:**

Dialysis adequacy, and patient and technique survival are similar in the ADPKD and non-ADPKD patients treated with PD. PD seems a feasible treatment option for end-stage renal failure in the course of ADPKD.

## Introduction

Autosomal dominant polycystic kidney disease (ADPKD) constitutes the most common genetic kidney disease in the world and affects 1 per 1000 subjects [1]. The number of people suffering from ADPKD in Poland could be estimated at about 30,000–40,000. Most of them progress to ESRD during their lifetime when renal replacement therapy (RRT) is required. According to the Report on the Renal Replacement Therapy in Poland, in 2011, ADPKD was the fourth leading cause of ESRD, with a prevalence rate of 8.6 %, following diabetes mellitus (DM), primary glomerulonephropathies, and arterial hypertension [2]. Similarly, in other European registries, ADPKD accounts for 4.2–12.6 % of all patients currently treated with RRT [3].

Over the past 20 years, due to improved survival of ESRD patients, the number of ADPKD patients receiving RRT markedly increased [4]. As in other nephropathies, the decision on the type of dialysis modality in patients with ADPKD is usually based on the patient’s choice, physician’s experience, or preferences, as well as resource availabilities.

Faced with the decision on the best dialysis option, many physicians are reluctant to choose peritoneal dialysis (PD), due to the potential disadvantages of the method in this patients’ population: risk of hernias and fluid leakages, risk of peritonitis and cyst infection, or insufficient dialysis adequacy. However, most studies reporting the risk of the above-mentioned complications have not provided even estimated data on the incidence or prevalence. Thus, the exact risk is largely unknown. Depending on the source, peritoneal leakage episodes were reported in 14.2 or 15 % of the ADPKD patients, but varied from 0.25 to 11.8 % in non-ADPKD groups [5, 6]. Reported abdominal wall hernias needed surgical intervention, but there were no differences in the requirement for temporary use of HD in both groups. All ADPKD patients with abdominal hernias were reported to be able to resume PD after surgical repair [7].

The concept of an integrated approach to RRT assumes that in most cases, PD ought to be considered as a treatment option. However, in many centers, this is not the case for patients with ADPKD. This is mainly due to concerns for the possible impaired dialysis efficacy due to enlarged kidneys that potentially reduce the available effective peritoneal surface area [8]. Furthermore, the risk for abdominal wall hernias and peritoneal leaks is thought to be increased. Finally, owing to an increased incidence of diverticulitis in ADPKD, the risk of peritonitis, especially with the Gram-negative bacteria, might be augmented. Whether these threats outweigh benefits of PD in ADPKD patients is debatable. According to recent data, adjusted mortality decreased by 45 % in ADPKD patients starting PD, while it decreased by only 25 % in those starting HD during the last 20 years [4].

The aim of the present study was to clarify whether the efficacy and the course of PD therapy in patients with ADPKD varies from that observed in subjects with ESRD due to other nephropathies, based on the data from the Polish Peritoneal Dialysis Registry (PPDR).

## Methods

We conducted a cross-sectional survey based on the data from the PPDR, which included information on subjects treated with PD in 63 dialysis centers all over the country. The registry acts under the Polish Society of Nephrology and data are voluntarily put in a computer database. Data are recorded at the initiation of PD and every year thereafter.

The study cohort consisted of 2394 incident PD patients. The inclusion period was between 2006 and 2013. For the present analysis, patients underwent a follow-up for a maximum of 5 years from the start of dialysis until death, technique failure or censoring. The data obtained from the PPDR included patient’s age, gender, primary renal disease, comorbidities, basic laboratory results, as well as data on the medication used, methods, and adequacy of dialysis. Hypertension and DM were comorbidities collected in the registry. Hypertension was defined as a blood pressure >140/90 mmHg or if the patient was taking antihypertensive medications. DM was diagnosed if patients were using insulin or oral hypoglycemic agents or if the fasting glucose concentration was >126 mg/dL, twice. Laboratory values collected in the registry comprised blood levels of hemoglobin (g/dL), albumin (g/L), calcium (mg/dL), and phosphorous (mg/dL). Dialysis adequacy was assessed using 24-h dialysate and urine collection with a calculation of total weekly Kt/V. Peritoneal transport was assessed using the standard peritoneal equilibration test. Of all the repeated measures in an individual patient (laboratory values, dialysis adequacy, etc.), the most recent were used for the analysis. For comparisons, the weekly dose of erythropoiesis-stimulating agents (ESA) in patients not taking erythropoietin-beta was converted into erythropoietin-beta units.

Patients included in this study were all subjects over 18 years of age, recorded in PPDR and treated with chronic PD due to end-stage renal disease. Patients were included in the ADPKD group based on the registry records, and diagnosis of the disease was left for the discretion of the attending physician. Exclusion criterion was the lack of a definite diagnosis of the underlying kidney disease.

The outcomes examined were technique failure, survival, and occurrence of complications: peritonitis episodes, tunnel infections, exit-site infections, hernias, and peritoneal fluid leakages.

Technique failure was defined as a permanent cessation of PD due to PD-related complications. For the technique survival analysis, patients were censored at transplantation, death, recovery of renal function, or when completing the follow-up period. Technique survival status was censored at the end of the follow-up period, i.e., September 12, 2014. Survival was determined from the initiation of PD treatment, and the patients were followed for a maximum of 5 years, with a median follow-up period of 32 months. For the patient survival analysis, subjects were censored at transplantation, transfer to HD, recovery of renal function, or when completing the follow-up period. Survival status was censored on September 12, 2014.

The diagnosis of peritonitis was made on the basis of white blood cell count >100/µL in peritoneal fluid effluent or Gram-positive stain or bacterial culture from the effluent. Exit-site infection was defined according to the standard scoring by Twardowski [9]. Tunnel infection was defined clinically as the presence of tenderness and induration or ultrasonographically as an evidence of fluid collection along the catheter.

### Statistical analysis

Results are expressed as mean and standard deviation or median and interquartile range, as appropriate. A *P* value <0.05 was considered to be statistically significant. Comparisons between two groups were assessed for continuous variables with a Student’s unpaired *t* test, or Mann–Whitney test, as appropriate. For categorical variables, a Chi-square test was utilized.

Survival analyses were made with the Cox proportional hazard model. The relative risks for mortality were determined by multivariate Cox regression analysis and presented as hazard ratios [hazards ratio (HR); 95 % confidence intervals (CI)]. The statistical analysis was performed using statistical software Statistica version 7.1 (StatSoft Inc.).

## Results

The study population consisted of 2394 patients included in the registry between 2006 and 2013. From this cohort, we excluded 682 patients because of the lack of diagnosis or the diagnosis not established. Patient’s demographic and baseline characteristics were not significantly different between excluded and further analyzed group.

Thus, we analyzed data of 1712 patients, where 106 patients were diagnosed with ADPKD, and the remaining group of 1606 subjects consisted of patients with a diagnosis of diabetic nephropathy (33.3 %), primary glomerulonephritis (26.8 %), hypertensive nephropathy (11.8 %), tubulointerstitial nephritis (11.6 %), and other (16.5 %). The general characteristics of the ADPKD and the non-ADPKD subjects are presented in Table 1. Patients suffering from ADPKD were, on average, older and had a greater proportion of women, as compared to the non-ADPKD group. The basic laboratory parameters, as well as the dialysis adequacy and peritoneal membrane permeability, did not differ significantly between the groups. Information on dialysis-associated complications was reported by 18 PD centers. These patients (*N* = 732) did not differ significantly from the other participants in terms of the basic clinical characteristics (not shown). The data on the dialysis-associated complications in this subset of analyzed patients are presented in Table 2. The risk of peritonitis was comparable in the two groups, as was the risk for other complications. It has to be stressed that the risk of abdominal hernias and of dialysis fluid leaks was twice as high in the ADPKD group, as compared to the controls, and did not reach statistical significance mainly due to the low numbers of patients affected by these complications.

**Table 1:** General characteristics of the autosomal dominant polycystic kidney disease (ADPKD) and non-autosomal dominant polycystic kidney disease patients treated with peritoneal dialysis

	ADPKD (*n* = 106)	Non-ADPKD (*n* = 1606)	*P* value
Age (years)	62 (55–72)	60 (47–71)	0.04
Gender (% male)	42.5	53.3	0.05
Mean time PD treatment (months)	44.3	46.3	0.6
PD modality (% of APD)	45.3	44.4	0.9
Renal transplantation (%)	26.4	18.6	0.04
Transferred to HD (%)	18.9	21.4	0.5
Renal function recovered (%)	0	1.2	0.3
Superimposed diabetes mellitus (%)	11.3	9.6	0.6
Hypertension (%)	83.0	83.9	0.9
Albumin (g/L)	37.3 ± 6.0	36.0 ± 7.3	0.1
Ca × P	46.9 ± 13.6	45.9 ± 14.1	0.7
Hemoglobin (g/dL)	11.4 ± 1.5	11.2 ± 1.7	0.2
Need for ESA (%)	73.6	75.2	0.7
Mean ESA dose (U/week)	3351 ± 1918	3524 ± 2159	0.5
Kt/V	2.22 ± 0.59	2.33 ± 0.71	0.2
D:P creatinine (4 h)	0.65 ± 0.13	0.68 ± 0.14	0.1

*ADPKD* Autosomal dominant polycystic kidney disease, *APD* automated peritoneal dialysis, *D:P* dialysate/plasma index, *ESA* erythropoiesis-stimulating factor, *PD* peritoneal dialysis

**Table 2:** Dialysis-associated complications in the autosomal dominant polycystic kidney disease (ADPKD) and non-autosomal dominant polycystic kidney disease patients

	ADPKD (*n* = 37)	Non-ADPKD (*n* = 695)	*P* value
Peritonitis	25	464	0.8
Peritonitis rate (episode per patient months)	1 per 32	1 per 25	0.8
Tunnel infection	2 (5.4 %)	23 (3.3 %)	0.8
Exit-site infection	3 (8.1 %)	58 (8.3 %)	0.8
Hernia	2 (5.4 %)	16 (2.3 %)	0.5
PD fluid leak	3 (8.1 %)	26 (3.7 %)	0.4

All data reflect the number of patients

The maximal follow-up time was 5 years with a median observation period of 32 months. Since the groups were different with respect to age and gender, we performed a Cox proportional hazard analysis which included these and other potential confounders (Table 3). It demonstrated that ADPKD was not associated with a different risk for poor outcome in comparison with the other nephropathies. Mortality rates were 5.8 deaths/100 patient-years in ADPKD and 6.3 in non-ADPKD group. Technique survival was also evaluated with the Cox proportional hazard analysis, as demonstrated in Table 4. The overall technique failure rates were 18.9 and 21.4 % in ADPKD and non-ADPKD group, respectively. As with the patient survival, ADPKD showed absolutely no associations with the risk of technique failure.

**Table 3:** Cox proportional hazard analysis for all-cause mortality (*n* = 1712; ADPKD 106; non-ADPKD 1606)

	HR (95 % CI)	*P* value
ADPKD	0.76 (0.41–1.40)	0.4
Age	1.05 (1.04–1.06)	<0.001
Gender	1.10 (0.84–1.44)	0.5
Diabetes mellitus	1.54 (1.18–2.01)	0.002
Hypertension	0.50 (0.37–0.70)	<0.001

**Table 4:** Cox proportional hazard analysis for peritoneal dialysis technique failure (*n* = 1712; ADPKD 106; non-ADPKD 1606)

	HR (95 % CI)	*P* value
ADPKD	1.04 (0.64–1.68)	0.9
Age	1.00 (0.99–1.00)	0.4
Gender	0.81 (0.64–1.01)	0.07
Diabetes mellitus	1.09 (0.86–1.39)	0.5
Hypertension	1.30 (0.92–1.84)	0.14

Since the presence of DM turned out to be an independent predictor of all-cause mortality, we repeated all the above-mentioned analyses following exclusion of diabetic patients. However, the results concerning the clinical and laboratory data, as well as the outcome, did not change substantially (not shown). Also, we found no difference in the risk of dialysis-associated complications or the treatment outcome between APD and CAPD subjects.

## Discussion

The present study, based on a large national registry, evaluates mortality and technique survival in a relatively large ADPKD population treated with PD. It reveals that the dialysis adequacy, represented in this study by weekly urea Kt/V, calcium phosphate product, as well as by hemoglobin levels, and the need for ESA, is not different in the ADPKD patients, as compared to the non-ADPKD controls. Furthermore, the patient and technique survival is similar in the ADPKD and non-ADPKD subjects. The results argue against the above-mentioned concerns related to the applicability of PD in the ADPKD population. Moreover, they stay in accordance with some previous reports in other ADPKD cohorts. In a study by Kumar et al. [10], PD therapy long-term outcomes were identical in patients with ADPKD and in non-diabetic matched controls. In a Chinese cohort of 42 consecutive ADPKD patients, the 5-year patient and technique survival was not different from that of 84 matched non-ADPKD controls [7]. Also, on the basis of the results of the multicenter prospective matched-cohort study of 106 ADPKD patients and 312 controls, Janeiro et al. [6] have concluded that PD is a suitable RRT option in ADPKD. On the other hand, we should take into account that some selection bias might occur, as patients with an advanced organomegaly might not be qualified for PD in most centers. Recently, Hamanoue et al. [11] have evaluated the influence of kidney and liver volumes on continuation of PD in patients with ADPKD. They have concluded that PD performance may be limited in patients with very enlarged organs, due to abdominal hernias and leaks of dialysis fluid. Clearly, this shows a need for a large registry collecting data on total kidney volume, liver volume, cyst infections, etc.

In our observation, the risk of abdominal hernias and dialysis fluid leaks was twice as high in the ADPKD groups than in the other patients, a difference that did not reach statistical significance due to the low number of events, but surely is of clinical importance. These results stay in accordance with other studies confirming that these complications constitute a considerable problem in ADPKD PD patients [7, 11–13]. However, both our study and the previous evaluations document that in a vast majority of patients, hernias and fluid leaks are treatable, as they are not associated with a permanent transfer to hemodialysis. It has to be underlined that the number of hernias and leaks was relatively low in our study. Similar results were obtained in a study by Hadimeri et al. [14] in which the risk of developing hernia was, similarly, twice as high in ADPKD patients, but the difference did not reach statistical significance. Other studies showed similar proportions but higher rate of these complications [7].

The risk of other PD-related complications, especially peritonitis, was similar in both groups. Concerns for the potentially higher risk of peritonitis in ADPKD patients treated with PD have originated from the fact that diverticulitis is relatively common in the course of this disease. However, the studies performed to date show similar results to the present evaluation, with no difference in the peritonitis rate between ADPKD and non-ADPKD groups [7, 15, 16].

We found no difference in the proportion of PD modalities (APD vs. CAPD) in ADPKD patients as compared to non-ADPKD group. Due to reduced intraperitoneal pressure in APD technique, this method of treatment seems to be preferred in ADPKD individuals in some centers [16]. In a recent report by Janeiro et al. [6], the percentage of ADPKD patients treated with APD has been no different as compared to other nephropathies.

We have expected the dose of ESA to be lower in ADPKD, as synthesis of erythropoietin in polycystic kidneys is maintained even at late stages of the disease. It has proven not to be the case as there has been no difference between the groups in this respect.

The major limitation of the present study is the retrospective nature of the current analysis. We also have to state that our study is confirmatory in nature, as studies have already been conducted in this field, as discussed above. Nevertheless, in our opinion, these data from a large national cohort add to our understanding of the evaluated issue. Another potential limitation is that the evaluation is based on a registry data, which might be influenced by underreporting. However, even if it had been the case, it would have affected both the ADPKD and non-ADPKD patients. Therefore, it should not bias the relative comparisons. Our data on peritonitis do not include the pathogen identification. Thus, we were unable to assess the rate of peritonitis episodes caused by Gram-negative bacteria, potentially more prevalent in the course of diverticulitis. Finally, although the data were based on a national registry, the number of subjects was still rather low, resulting in a lack of sufficient statistical power to identify certain clinically meaningful differences between the groups, as, for instance, the risk of abdominal hernias. It should be noted that on the basis of the registry data, we are not able to provide and analyze additional information regarding PD complications, as, for example, the information on kidney and liver volume, or the influence of the time of commencement of PD following catheter placement on the occurrence of peritoneal leakages. Selection bias should be also considered, as PD might not be offered to a proportion of ADPKD patients due to some actual or potential contraindications. The observation time of a maximum 5 years might be regarded as rather short, although with a high mortality rate typical for dialysis patients, as well as a high dropout due to transplantations, it is sufficient, in our opinion, for reliable conclusions.

According to the ERA–EDTA Registry, the relative contribution to RRT of PD equals 5.8 % in ADPKD patients, while it is as high as 7.1 % in the non-ADPKD subjects [4]. Simultaneously, the increase in the transplantation rate has been noted in this group [4]. The relatively lower prevalence of PD in ADPKD might be due to the above-mentioned concerns. Although most probably it is a consequence of the higher proportion of transplanted patients among the ESRD subjects with ADPKD, compared to patients with other nephropathies, it still shows the degree of underutilization of PD treatment in the ADPKD population. Unfortunately, this trend can be also observed in our country. According to the Report on the Renal Replacement Therapy in Poland, the proportion of ADPKD patients treated with PD constituted only 4 % of dialyzed subjects in 2011 [3]. The present study demonstrates that the dialysis adequacy, and patient and technique survival are similar in the ADPKD and non-ADPKD patients treated with PD. PD seems a feasible treatment option for end-stage renal failure in the course of ADPKD and should be always considered as an important element of an integrated therapeutic approach.
